# Improving Self-Healing Dental-Restorative Materials with Functionalized and Reinforced Microcapsules

**DOI:** 10.3390/polym16172410

**Published:** 2024-08-24

**Authors:** Bao Quoc Huynh, Sivashankari Rajasekaran, Joao Batista, Steven Lewis, Mario Alexandre Coelho Sinhoreti, Carmem Silvia Pfeifer, Ana Paula Fugolin

**Affiliations:** 1 Division of Biomaterial and Biomedical Sciences, Department of Oral Rehabilitation and Biosciences, Oregon Health & Science University, Portland, OR 97201, USA; huynhb@ohsu.edu (B.Q.H.);; 2 Dental Materials Division, Department of Restorative Dentistry, Piracicaba Dental School, State University of Campinas (FOP-UNICAMP), Piracicaba 13414-903, SP, Brazil

**Keywords:** chemical functionalization, dental resin composites, melamine, microcapsules, poly(urea–formaldehyde), poly(urea–melamine–formaldehyde), self-healing dental composites, silane, surface chemical modification

## Abstract

Dental resin composites are widely used in clinical settings but often face longevity issues due to the development and accumulation of microcracks, which eventually lead to larger cracks and restoration failure. The incorporation of microcapsules into these resins has been explored to introduce self-healing capability, potentially extending the lifespan of the restorations. This study aims to enhance the performance of self-healing dental resins by optimizing the microcapsules–resin matrix physicochemical interactions. Poly(urea–formaldehyde) (PUF) microcapsules were reinforced with melamine and subsequently subjected to surface functionalization with 3-aminopropyltriethoxysilane (APTES) and (3-mercaptopropyl)trimethoxysilane (MPTMS). Additionally, microcapsules were functionalized with a bilayer approach, incorporating tetraethyl orthosilicate (TEOS) with either APTES or MPTMS. X-ray photoelectron spectroscopy (XPS) and thermogravimetric analysis (TGA) confirmed an increased Si:C ratio from 0.006 to 0.165. The functionalization process did not adversely affect the structure of the microcapsules or their healing agent volume. Compared to PUF controls, the functionalized microcapsules demonstrated enhanced healing efficiency, with TEOS/MPTMS-functionalized microcapsules showing the highest performance, showing a toughness recovery of up to 35%. This work introduces a novel approach to functionalization of microcapsules by employing advanced silanizing agents such as APTES and MPTMS, and pioneering bilayer functionalization protocols through their combination with TEOS.

## 1. Introduction

Since methacrylate-based dental resin composites were introduced in the market in the early 1960s, they have undergone dramatic improvements, establishing them as the most popular restorative material in clinical practice [1]. Despite their enhanced physicochemical properties that enable them to withstand the harsh oral environment, the longevity of resin-based dental restorations is still suboptimal, with 60% of restorations requiring replacement within the first 10 years [2,3]. The replacement of dental restorations imposes a significant economic burden and, more importantly, almost inevitably leads to the removal of sound dental tissues, weakening the tooth structure [4]. This weakening diminishes the prognosis of direct restorations and often leads to more aggressive and complex indirect restorative procedures [4].

The most common reasons for the replacement of dental restorations are recontamination of dental tissues and material fracture [5,6]. In both cases, the development of microcracks due to masticatory forces and thermal fluctuations plays a crucial role [5,7]. These microcracks typically form and propagate until they merge, leading to macroscopic structural failure [8]. Various strategies have been explored to prevent this process, inspired by advances in other fields; self-healing synthetic dental materials have emerged as a promising approach to address crack propagation at an early stage [9].

Considering the manufacturing process of dental resin composites and their highly crosslinked, high glass transition temperature (Tg) polymeric networks, the incorporation of microcapsules loaded with a polymerizable, low-viscosity healing agent has shown promise in enhancing the longevity of resin-based dental restorations [10,11]. In this approach, microcapsules embedded in dental resin composites release their healing agent when microcracks propagate through the resin matrix [11]. The microcracks rupture the polymer shell of the microcapsules, allowing the healing agent to flow into the damaged area [11]. Upon reaction with benzoyl peroxide (BPO) dispersed in the dental resin, the tertiary amine-containing healing agent polymerizes, effectively sealing the microcracks [11]. Poly(urea–formaldehyde) (PUF) is frequently used for these microcapsules [11,12], encapsulating various cargoes such as TEGDMA, BISGMA, and DMAM [11,13].

Although this approach has demonstrated promising results, two significant limitations have constrained its further development: the ratio of microcapsules that can be incorporated into formulations and the potential for shell integrity issues, where cracks may propagate without causing rupture [11]. The limitation in incorporating a higher ratio of microcapsules is due to their lack of chemical bonding with the organic matrix, causing them to behave as voids or defects, which compromise the material’s mechanical properties at higher concentrations [14]. The non-rupture of microcapsules is mainly due to their mobility, which allows microcracks to deflect away, propagating through the shell-organic matrix interface that offers less resistance [15]. Both issues could be addressed by creating chemical interactions between the microcapsule shells and the organic matrix, utilizing the abundance of amino and hydroxyl groups in the poly(urea–formaldehyde) network to attach functionalizing agents, thereby integrating microcapsules and the organic matrix into a cohesive structure.

Therefore, this study aims to develop, synthesize, and test a broad array of functionalization protocols to enhance the performance of microcapsules designed for self-healing dental polymers. Three different functionalizing agents were selected and tested individually or in combination. 3-aminopropyltriethoxysilane (APTES) was chosen due to its extensive use in biological applications [15]. In PUF polymers, APTES’s amino groups have demonstrated the capability to scavenge free formaldehyde molecules, further improving the biocompatibility of these systems [16]. Additionally, the amino groups can react with methacrylate functionalities, forming β-amino ester-based networks [17]. 3-mercaptopropyltrimethoxysilane (MPTMS) was selected to leverage the chain transfer reactions performed by pendant thiols, which have proven advantageous during the polymerization of methacrylates by creating an environment that is less conducive to the development of polymerization stress [18,19]. Lastly, tetraethyl orthosilicate (TEOS) was selected as a preliminary treatment agent to increase the free hydroxyl groups on the microcapsule surface [20]. Its association with APTES and MPTMS has been shown to lead to greater crosslinking during surface functionalization, as compared to a single silane [21]. To increase the stability of the microcapsules and retention of the healing agent volume during the functionalizing procedures, the reinforcement of the poly(urea–formaldehyde) network with melamine was tested. This strategy has been widely tested in other fields and resulted in shells with high mechanical properties [15]. The tested hypothesis is that the surface modifications imparted by the proposed functionalization protocols will result in greater self-healing efficiency compared to PUF microcapsules.

## 2. Materials and Methods

Triethylene glycol dimethacrylate (TEGDMA, 95%), poly(ethylene maleic anhydride) (pEMA; M.W: 100,000 to 500,000) (pEMA), resorcinol (ACS Reagent ≥ 99%), 4-(Dicyanomethylene)-2-methyl-6-(4-dimethylaminostyryl)-4H-pyran (DCM; dye content 98%), diurethane dimethacrylate (UDMA; ≥97%), bisphenol A ethoxylate dimethacrylate (BisEMA), and phenylbis(2,4,6-trimethylbenzoyl)phosphine oxide (BAPO, 97%) were procured from Sigma Aldrich (St. Louis, MO, USA). N, N-Dimethylacrylamide (DMAM; 99% purity) was obtained from Acros Organics (Geel, Belgium) and urea (Ultrapure™ Urea) from Invitrogen (Waltham, MA, USA). Ammonium chloride, formaldehyde (37%), and sodium meta-periodate (certified ACS) were obtained from Fisher Scientific (Hampton, NH, USA). (3-aminopropyl) triethoxysilane (APTES; 97%), tetraethyl orthosilicate (TEOS; 90%), benzoyl peroxide (BPO; 75%), and 2,2′-(p-Tolylazanediyl)diethanol (DHEPT; 95%) were obtained from Oakwood Chemical (Estill, SC, USA). (3-Mercaptopropyl) trimethoxysilane (MPTMS; >96.0%) was obtained from TCI America (Portland, OR, USA). 4-Amino-3-hydrazino-5-mercapto-1,2,4-triazole (Purpald; 99 + %) was obtained from Alfa Aesar Chemicals (Heysham, Lancashire, UK). All reagents were used without any additional purification.

### 2.1. Synthesis of Poly(Urea–Melamine–formaldehyde) (PUMF) and Poly(Urea–Formaldehyde) (PUF) Microcapsules

The PUMF microcapsules were prepared by an oil-in-water double-emulsion technique following procedures previously described [13,22], based on the standard synthetic steps for formation of poly(urea–formaldehyde) networks [13,23,24]. Briefly, 50 mL of distilled water was mixed with 13 mL of a 2.5% (*w*/*v*) solution of pEMA in a 250 mL 3-neck round-bottom flask. The reaction mixture was heated up to 50 °C and stirred with a stainless-steel impeller (blade width = 4.5 cm) at 400 rpm. To the flask, 0.125 g of ammonium chloride, 0.125 g of resorcinol, 0.22 g of melamine, and 1.25 g of urea were added and stirred until all reagents were dissolved. The pH of the reaction mixture was increased from 2.7 to 3.5 via the dropwise addition of 1 M NaOH. Separately, 24 g of TEGDMA, 6 g of DMAM, and 0.06 g of DCM dye were mixed. The healing agent system was tagged with the laser dye DCM to assist with microcapsules characterization and healing agent stability monitoring. The resulting core solution was then added dropwise to the reaction mixture to form microdroplets. The reaction mixture was allowed to stir for 10 min to stabilize the emulsion; then, the temperature was raised up to 55 °C. Once the temperature was stable, 3.15 mL of 37% formaldehyde was added dropwise to the mixture. The reaction mixture was subjected to thermopolymerization for 4 h. After this period, the suspension of microcapsules was removed from heat and allowed to cool to room temperature. The contents of the reaction flask were transferred into a beaker containing 500 mL of distilled water and allowed to settle for 30 min before the decanting of the microcapsules. The supernatant containing unreacted starting materials was removed and the microcapsules were rinsed with distilled water, vacuum-filtered, and then washed with 200 mL of hexanes and 100 mL of chloroform. In the final synthetic step, microcapsules were dispersed on a watch glass and dried at room temperature for 24 h. Non-melamine PUF capsules were synthesized according to the same procedures described above, with the omission of mealime, and tested as the control [13].

The success of the synthetic procedures was confirmed by analyzing the microcapsules using optical microscopy at 4× and 10× magnification with AmScope Trinocular Compound Microscope (Model BS1153-EPL, AmScope, Irvine, CA, USA).

### 2.2. Surface Functionalization of PUMF Microcapsules

Five different experimental shell surface treatments were tested to promote PUMF microcapsule functionalization and/or reinforcement. These treatments included the following: (1) functionalization with APTES silane agent, (2) functionalization with thiol-based MPTMS silane agent, (3) reinforcement with a silica coating using TEOS, (4) combination of TEOS coating and APTES functionalization, and (5) combination of TEOS coating and MPTMS functionalization. PUMF and PUF non-functionalized microcapsules were tested as controls. Microcapsule stability in different solvent systems was also assessed to determine the appropriate solvent for functionalization.

#### 2.2.1. Functionalization with APTES or MPTMS: Monolayer Silane Functionalization

For these groups, a 4% *w*/*w* solution of either APTES or MPTMS in water (pH = 3) was prepared, based on protocols previously described in the literature in other fields [10,15,25,26,27]. Microcapsules were added to the silane solution at 1% with respect to the mass of the silane solution and stirred in a beaker with a stainless-steel impeller (blade width = 4.5 cm) at 400 rpm and at room temperature. After 24 h, the microcapsules were allowed to settle for 30 min before decanting the supernatant. The microcapsules were then washed with water, hexanes, and chloroform, dispersed on a watch glass, and dried at room temperature for 24 h.

#### 2.2.2. Silica Coating Reinforcement with TEOS

The silica coating protocol followed procedures described in the literature for TEOS functionalization in other fields [21,28]. In the first step, a 4% *w*/*w* aqueous solution of TEOS (pH = 3) was prepared. Similarly, microcapsules were added to the silane solution at 1% by mass and stirred with the same conditions as previously described. After 12 h, the microcapsules were allowed to settle for 30 min for decanting, then washed, filtered, and dried, following the same procedures described above.

#### 2.2.3. Combination of Silica Coating Reinforcement with TEOS and Functionalization with APTES or MPTMS: Bilayer Silane Functionalization

For the combined reinforcement and functionalization surface treatments, microcapsules were first subjected to the TEOS treatment as described in Section 2.2.2. Following this, the microcapsules were resuspended in a 4% *w*/*w* solution of either APTES or MPTMS (pH = 3) and stirred at 400 rpm for an additional 12 h at room temperature. After this period, the microcapsules were allowed to settle; then, they were decanted and washed with water, hexanes, and chloroform. Finally, the microcapsules were dried at room temperature for 24 h.

### 2.3. Characterization of the Surface-Modified PUMF Microcapsules

#### 2.3.1. Morphological Characterization by Optical and Scanning Electron Microscopies (SEMs)

For monitoring the microcapsule’s structural integrity and healing agent stability, all the newly synthesized groups of microcapsules were analyzed by optical microscopy using a AmScope Trinocular Compound Microscope (Model BS1153-EPL, AmScope, Irvine, CA, USA). The microcapsules were dispersed in distilled water, pipetted onto a glass slide, and then imaged at 10× with a cover slip. Images were taken with the microscope camera.

For SEM analysis, the microcapsules were dispersed in distilled water and drop-casted onto a silicon wafer. After drying, the capsules were sputter-coated with gold–palladium using a Denton Vacuum Desk II sputter coater for 10 s. SEM micrographs were obtained using a FEI Quanta 200 in low-vacuum mode at 10 kV, with 500× and 1000× magnification, and a 20 mm working distance.

#### 2.3.2. Microcapsules Surfaces Functionalization Efficiency by Thermogravimetric Analysis (TGA)

For each experimental group, approximately 15 mg of microcapsules were placed in a platinum pan, equilibrated at 50 °C, and heated to 65 °C for 10 min. The microcapsules were then heated to 850 °C at a ramp rate of 20 °C/min (Discovery TGA55, TA Instruments) (n = 3). The percent mass loss was recorded as a function of temperature, and triplicate runs were averaged. Additionally, TGA data were obtained for TEGDMA, unfunctionalized PUF and PUMF, and all silanizing agents using the same method.

#### 2.3.3. Elemental Composition of the Microcapsules Surfaces by X-Ray Photoelectron Spectroscopy (XPS)

Microcapsules were prepared for XPS analysis by pressing approximately 4 mg into indium foil for charge mitigation. Monochromated Al Kα radiation (1486.6 eV) and ultra-high vacuum were used for obtaining the spectra (SPECS Ambient Pressure XPS). For all experimental groups, survey spectra were taken, as well as fine-scan spectra of the C^1s^, Si^2p^, and O^1s^ regions. Survey spectra were taken with a 100 eV pass energy, 1 eV/step, and 0.1 s dwell-time. For fine-scan spectra, 35 eV pass energy, 0.1 eV/step, and 0.1-0.4 s dwell-time were used.

### 2.4. Healing Efficiency of Microcapsule-Loaded Dental Resins

To assess the impact of the microcapsule surface treatments on the healing performances of dental resins, additional microcapsules were synthesized as per the previous procedure, with the addition of 0.3 g of tertiary amine DHEPT to the core solution to allow for chemical polymerization of the healing components once in contact with the BPO dispersed into the organic matrix. These new microcapsules were characterized and functionalized in the same manner as the non-DHEPT microcapsules. The synthesized microcapsules were included at 10 wt% in a dental resin formulation consisting of BisGMA:BisEMA:UDMA:TEGDMA (2:2:2:1) with 1 wt% BAPO and 0.5 wt% BPO.

For the healing efficiency assessment, fracture toughness bars for the single-edge notch beam method were prepared in accordance with ASTM E399-90 standards. The capsule-loaded dental resins were cast into 5 mm × 2 mm × 25 mm steel molds with a razor blade insert, resulting in bars with a 2.5 mm deep notch in the middle (n = 10). The bars were photocured with five overlapping photoactivation shots of 40 s on each side using an SDI Radii-cal at a radiant emittance of 1200 mW/cm^2^. After storing the bars in dry vacuum conditions for 24 h, excess material was removed by sanding with 1000-grit sandpaper. The samples were catastrophically fractured using a universal testing machine (MTS Criterion, Eden Prairie, MN, US) at a crosshead speed of 0.5 mm/min with a 100 N load cell, and the load required to fracture the bar was recorded in N (L1). Immediately after fracture, the split bars were re-joined using a stainless-steel fixture and allowed to heal at 37 °C for 24 h. After the healing period, the samples were subjected to a post-healing fracture toughness test, and the resulting loading was recorded (L2). Photographs were taken after fracturing and after the healing period using a 100 mm macro lens with a coupled flash (Canon EOS 60D). The healing efficiency (%) was calculated by dividing L2 by L1 and multiplying by 100.

The fractured surface of the resin bars was observed under SEM to characterize the crack morphology, propagation, and microcapsule behavior. Following the healing efficiency tests, representative fractured bars were cut into 5 mm tall pieces using a metallographic cutting machine. Subsequently, the samples were cleaned with water in an ultrasonic bath, were mounted on a metal stub with the fracture-side facing up using carbon tape and coated with a conductive adhesive (PELCO SEM-Gold/Silver Extender). A gold–palladium sputter coating was applied on the fractured surface for 15 s. SEM micrographs were captured in high-vacuum mode at 15 kV, with an average working distance of 25 mm and magnifications of 50× and 200×.

### 2.5. Statistical Analysis

For sample size calculations, a power analysis was conducted considering a power value of 0.8. This analysis was based on data obtained from a pilot test which were used to determine the appropriate sample sizes for assessing the diameter of capsules, performing TGA, and evaluating fracture toughness bars for healing efficiency. The analysis was performed using Origin(Pro) software, version 2024 (OriginLab Corporation, Northampton, MA, USA). All data were assessed for normality and homoscedasticity using Anderson–Darling and Levene tests, respectively. Statistical analysis was performed using one-way ANOVA followed by Tukey’s test for multiple comparisons (α = 0.05). Two-way ANOVA and Tukey’s test for multiple comparisons (α = 0.05) were conducted on the Purpald assay results, which will be detailed in the Results and Discussion section.

## 3. Results and Discussion

**Figure 1:**
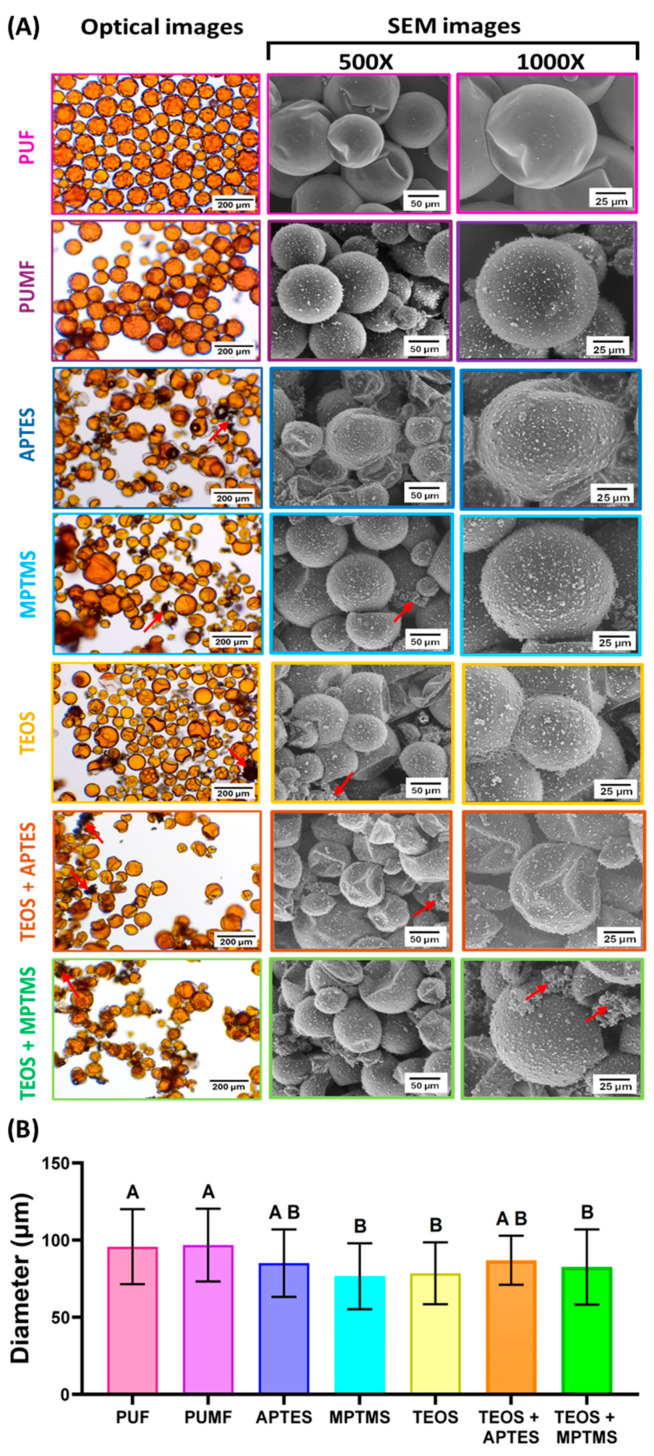
(**A**) Optical and scanning electron micrographs of unfunctionalized (PUF and PUMF) and PUMF-functionalized microcapsules. Optical micrographs were taken after dispersing the microcapsules in distilled water at 10× magnification. For SEM imaging, the microcapsules were dispersed on a silicon wafer plate, sputter-coated with gold, and imaged in low vacuum mode at 10 kV with an average working distance of 20 mm and magnifications of 500× and 2000×. Red arrows indicate the formation of aggregates of both silane and melamine–formaldehyde nanoparticles. (**B**) Average diameter (µm) of the microcapsules for all synthesized groups. The diameters were calculated by analyzing the optical micrographs using ImageJ, measuring, and averaging the diameter of 50 microcapsules for each group. Data from all groups were analyzed by one-way ANOVA and Tukey’s test; different letters indicate statistically significant differences (*p* < 0.05).

**Figure 2:**
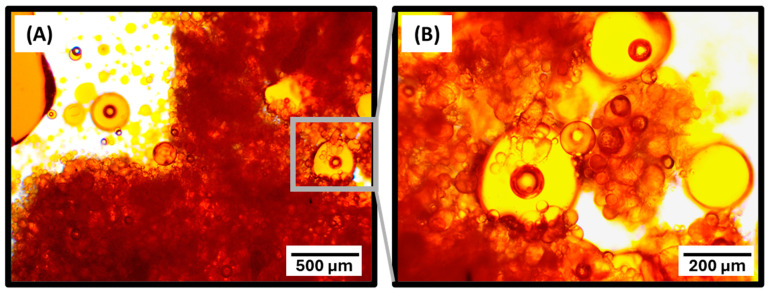
Optical micrographs of PUF microcapsules at 4× (**A**) and 10× (**B**) magnifications after treatment with the APTES functionalizing agent. Pronounced leakage of the healing agent (tagged in orange) is observed, attributed to the fragility of the microcapsule shells, which did not withstand the additional stirring and solvent exposure required for the surface functionalization procedures.

In this study, protocols for the functionalization and reinforcement of PUF microcapsules were designed, synthesized, and tested as strategies to increase stability and create chemical interactions between the shells and the organic matrix, enhancing the performance of microcapsules intended for self-healing dental polymeric materials. Given that the functionalization and reinforcement protocols involve exposing the microcapsules to additional shear forces and immersion in solvents, the structural integrity and volume of the healing agent within the microcapsule cores were carefully monitored. This is based on a previous study by our research group, which demonstrated that the shell polymeric network can act as a semi-permeable membrane when the capsules are dispersed in certain solutions, resulting in the influx of storage media and the efflux of cargo [13]. Optical microscopy was utilized to verify the morphology of the microcapsules and primarily to monitor the volume of the healing agent within the cores, ensuring that the surface modification protocols did not result in microcapsules partially filled with the healing agent. The optical micrographs for all the tested groups are presented in Figure 1A and they revealed microcapsules with preserved shell structural integrity and core retention, demonstrating that the designed synthetic protocols based on aqueous solutions, mild pH, and 24 h stirring are not harmful to the capsules. The structural integrity of the newly synthesized microcapsules was further confirmed by SEM micrographs (Figure 1A). Across all functionalized groups, some deformation of the microcapsules was observed. Although the spherical morphology was not completely preserved for the functionalized groups, optical microscopy indicated minimal to no loss of the encapsulated healing agent, as previously mentioned. The deformation of the microcapsules after drying may be attributed to the shrinkage of the shells due to the loss of residual solvents entrapped in the core [13]. Diameter measurements also highlighted shell shrinkage/deformation, showing a slight reduction (approximately 10 to 15%) in all experimental groups subjected to surface modification procedures compared to the untreated PUF and PUMF control groups (Figure 1B). It is important to note that functionalization of PUF capsules was attempted with APTES, but it resulted in significant loss of structural integrity of the microcapsules, massive healing agent leakage, and the formation of oily clumps (Figure 2). Therefore, melamine was incorporated as an additive into the poly(urea–formaldehyde) polymeric network to enhance its mechanical properties. Applications of melamine modified urea–formaldehyde resins are well documented and extensive in other fields, where they are selected for its greater strength, thermal stability, and reduced formaldehyde elution [29]. Melamine modification introduces a triazine ring into the polymeric structure, resulting in stronger crosslinking within the polymer network [30,31,32]. In fact, PUMF microcapsules presented superior retention of the core material than their PUF counterpart, resulting in uniform and free-flowing capsules. Therefore, the increased strength imparted by melamine addition was essential for preserving microcapsule integrity after functionalization. In addition, the differences in surface topographies between PUF and PUMF microcapsules are stark, as evidenced by the SEM micrographs (Figure 1A).

Overall, PUMF microcapsules exhibit a dramatic increase in surface roughness, which is mainly attributed to the formation and deposition of pre-polymerized urea–melamine–formaldehyde nanoparticles on their outer shells, making the microcapsule surface coarser [25]. While the exact bonding mechanism between the nanoparticles and melamine-containing shells is not entirely clear, it may involve amino groups that participate in the following formations: (1) covalent bonds via methylene bridges with the formaldehyde groups; and/or (2) hydrogen bonding with the carbonyl groups [33,34]. Additionally, it has been shown that amino groups in melamine can assist in forming an electrostatic layer [35] likely contributing to the attraction and binding of nanoparticles to the microcapsule shells. In PUMF-functionalized microcapsules, the surface roughness increased even further, which may be likely due to the additional deposition of silane [15]. Under optical microscopy, larger aggregates of both silane and melamine–formaldehyde nanoparticles appear as black, opaque particles, as seen in the micrographs of all silane-functionalized groups (red arrows in Figure 1A). Under SEM at 1000× magnification, these particles are observed as small, irregularly shaped particles, particularly prominent in the TEOS/MPTMS group.

**Figure 3:**
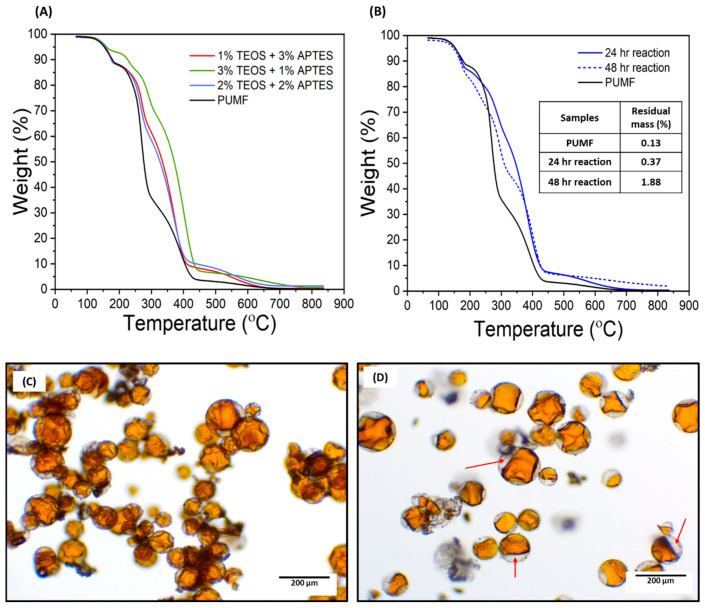
Thermogravimetric curves of functionalized microcapsules. Each sample was equilibrated at 50 °C, then heated to 65 °C and held for 10 min. Samples were subsequently heated to 850 °C at a ramp rate of 20 °C/min. (**A**) Thermogravimetric curves of microcapsules functionalized with TEOS and APTES at different ratios in a one-step process totaling 4 wt% to prevent compound precipitation. For all compared groups, capsules were functionalized in a single solution containing both TEOS and APTES. Testing with 4% TEOS and 4% APTES as a single solution preparation led to significant silane precipitation. (**B**) Comparison of microcapsules functionalized with TEOS and APTES in a two-step process with reaction times of 24 h and 48 h. “Two-step” groups were first functionalized with TEOS, then decanted, and subsequently functionalized with APTES. (**C**,**D**) Optical micrographs of PUMF microcapsules functionalized with TEOS and APTES in a two-step process: 12 h for each step (total 24 h reaction) (**C**) and 24 h for each step (total 48 h reaction) (**D**). In the 48 h reaction, a significant loss of the healing agent is observed, with the microcapsules being only partially filled, as indicated by the red arrows.

**Figure 4:**
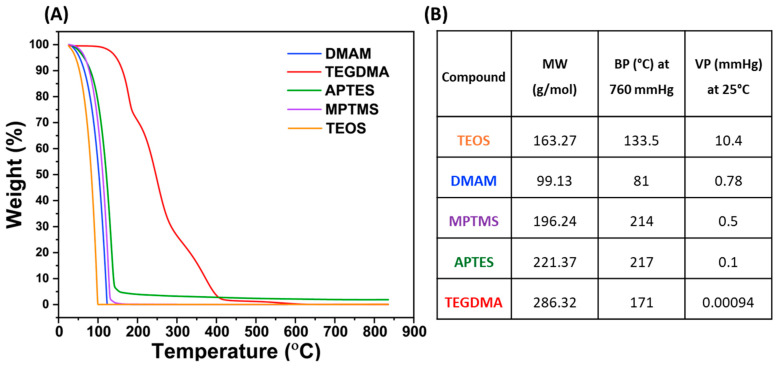
(**A**) Representative thermogravimetric curves of all the individual components of the microcapsules and functionalizing agents. (**B**) Table containing molecular weight (MW, g/mol), boiling point at 760 mmHg (BP, °C), and vapor pressure at room temperature (VP, mmHg) for all the starting materials. In general, the thermograms closely followed the vapor pressure of the materials. Specifically, the components with higher vapor pressure resulted in greater mass loss at lower temperatures.

It is worth mentioning that several different synthetic conditions were tested to maximize silane deposition while also minimizing overall damage to the microcapsules. The development of the synthetic protocols for the bilayer silane functionalization (TEOS/APTES and TEOS/MPTMS) was especially challenging since it required us to delicately balance the differences in reactivity between TEOS and the APTES/MPTMS without extending the stirring time. Initial testing of TEOS/APTES functionalization was performed with a single solution containing each one of the two silanes at 4% *w*/*w*, which resulted in precipitation of silane and gelling of the solution, indicating that the concentration of the silanes exceeded their solubility limit. Therefore, the concentration of both silanes was limited to 4% *w*/*w* and three different ratios were tested: 3% TEOS/1% APTES; 2% TEOS/2% APTES; and 1% TEOS/3% APTES. The thermograms acquired from TGA exhibited a more pronounced right shift in thermal degradation of capsules functionalized with 3% TEOS/1% APTES, but there was a negligible difference between the 1% TEOS/3% APTES and 2% TEOS/2% APTES groups (Figure 3A). TGA enables the study of the chemical and physical properties of materials by monitoring thermal changes as a function of increasing temperature and time [36]. To guide the analyses, the TGA data for all individual components were considered in light of their molecular weight, boiling point, and vapor pressure (Figure 4). These are important because they affect thermogram profile obtained in TGA assessments, which in turn reflects two key processes: the thermal decomposition of the material (chemical changes) and its evaporation rate (physical changes). Both of these result in measurable mass losses. Therefore, the curve shifts in comparison to the untreated PUMF thermograms and differences in the thermogram profiles were used as parameters to estimate the efficiency of each surface treatment. Overall, the three tested groups resulted in successful modification of the PUMF microcapsules, as evidenced by the differences in shapes of the thermograms compared to untreated PUMF microcapsules. The similarity between the 1% TEOS/3% APTES and 2% TEOS/2% APTES groups suggests that, essentially, only APTES was deposited on the microcapsule surface. This is attributed to the differences in reactivity between TEOS and APTES, where APTES, when dispersed in a single solution, outcompetes TEOS in binding to any free hydroxyl groups on the microcapsule surface. The lower reactivity of TEOS is likely associated with its slower rate of hydrolysis in comparison to APTES, due to the lack of electron-donating groups [37]. Electron-donating groups such as amino groups and thiol groups accelerate rates of hydrolysis in acidic media. Additionally, after each successive replacement of an ethoxy group with a hydroxyl group, the hydrolysis rates of the remaining ethoxy groups are further reduced due to electron-withdrawing effects [37]. This scenario favors TEOS deposition only when it is present in a much higher ratio than APTES in the reaction medium, as observed in the 3% TEOS/1% APTES group, which led to a more pronounced modification of the microcapsule surface, as evidenced by the more significant rightward shift of the thermogram. Therefore, to prevent the competition between TEOS and APTES in the reaction medium, which highly favored APTES and hindered double deposition, a two-step functionalization method was also tested. In this method, the microcapsules were first functionalized with TEOS, followed by a separate functionalization with APTES. This method also allowed to maintain the same 4% *w*/*w* concentration of silane agent in separate solutions, maximizing the available silane during functionalization. For the final optimization steps, 4% solutions of TEOS and APTES were used to test the impact of varying total reaction time. To compare the reaction times of previous single-layer functionalization protocols, the intervals of 24 and 48 h were selected. The TGA curves for the microcapsules showed a slightly more pronounced right shift in the thermal degradation curve of the 24 h capsules (Figure 3B). The 48 h capsules exhibited greater residual mass than the 24 h capsules, which is attributed to the formation of highly temperature-stable siloxanes [38,39]. However, optical micrographs revealed 48 h microcapsules were significantly more damaged than 24 h capsules, resulting in greater leakage of core material (Figure 3C,D). Therefore, considering the similarity in the thermal decomposition profiles between the 24 h and 48 h capsules, and to minimize structural damage to the microcapsules as well as core material loss, the reaction time for all bilayer-functionalized capsules was fixed at 24 h, using 12 h for each step.

**Figure 5:**
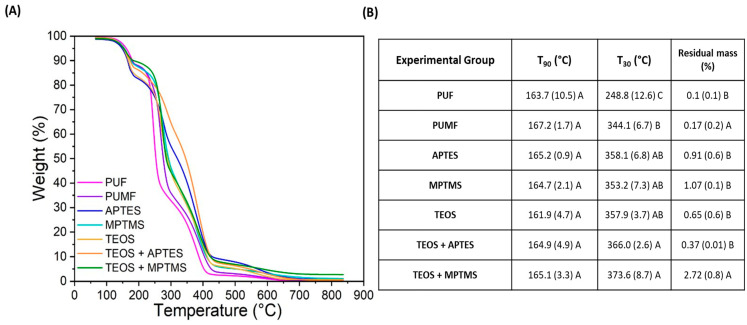
(**A**) Thermogravimetric curves of unfunctionalized and functionalized microcapsules. Each sample was heated to 850 °C at a ramp rate of 20 °C/min. Analyses for each group were conducted in triplicate, averaged, and plotted as curves. (**B**) Table depicting T90 (temperature at which the samples lost 10 wt% of their mass), T30 (temperature at which the samples lost 70% of their mass), and residual mass after the test, which accounts for the silica formation that is not thermally decomposed at temperatures up to 850 °C.

The efficiency of the functionalization procedures introduced in this study was carefully validated using TGA and XPS analyses. In the case of microcapsules, which, unlike glass filler particles, are completely degraded during the test, this analysis is more complex and serves better as a qualitative rather than a quantitative method. The thermal decomposition profiles of each group were analyzed in triplicate to assess the changes in thermal properties induced by each functionalization, and the resulting average curves are depicted in Figure 5A. Temperatures at which capsules lost 10% of their mass (T_90_) and 70% of their mass (T_30_) were selected as reference values for discussion of their thermal decomposition. The first noticeable difference is that all PUMF microcapsules exhibited a right shift in their thermogram curves compared to PUF and loss of 70% of their mass occurring at much higher temperatures (344.13 °C vs. 248.84 °C for PUMF and PUF, respectively) (Figure 5B). The increased thermal resistance observed in melamine-modified capsules was anticipated due to the higher crosslinking density facilitated by the triazine ring in the chemical structure of the melamine molecule [30,31]. Additionally, all the reinforcement/functionalization protocols resulted in a rightward shift of the thermograms compared to the non-functionalized PUMF control. This immediate effect strongly suggests that the synthetic protocols developed in this study effectively modified the surfaces of the microcapsules, thereby altering their chemical and physical properties. TEOS-modified microcapsules exhibited loss of 10% of their mass at the lowest temperature (T_90_ = 161.94 °C), which is likely related to the high vapor pressure of TEOS. However, when TEOS is combined with APTES or MPTMS, there is no significant difference in T90 compared to microcapsules functionalized solely with APTES or MPTMS (T_90_ for APTES = 165.18 °C, MPTMS = 164.66 °C, TEOS/APTES = 164.93 °C, and TEOS/MPTMS = 165.07 °C).

This similarity can be attributed to the two-step synthetic protocol, where microcapsules are first coated with TEOS followed by APTES or MPTMS. Therefore, the thermal decomposition behavior of the external layers in both single- and double-functionalized microcapsules, which are initially exposed to heat, is similar. In all functionalized microcapsules, the second mass loss event (T_30_) occurred at higher temperatures than the PUMF non-functionalized control, ranging from an increase of 9.04 °C (for MPTMS) to 29.46 °C (for TEOS/MPTMS). Notably, TEOS/APTES microcapsules retained 68% of their mass up to 300 °C. This enhancement in thermal resistance is likely due to the effects provided by the silane coupling agent molecules: (1) increase in crosslinking density and (2) formation of Si-O bonds [39,40]. The increase in crosslinking density alone makes the material more resistant to thermal degradation, as the reduced mobility of the polymeric chains poses a challenge for thermal energy to cause chain scission [38]. Si-O bonds, being more resistant to thermal cleavage than most organic bonds, further contribute to this stability [41,42]. Furthermore, the functionalized groups showed residual mass from 0.37% to 2.72% for TEOS/APTES and TEOS/MPTMS groups, respectively. This residual mass, resistant to temperatures as high as 850 °C, may be attributed to the formation of high-molecular-weight polysiloxanes [42]. These results collectively demonstrate that the tested protocols successfully modified the surface of the microcapsules.

**Figure 6:**
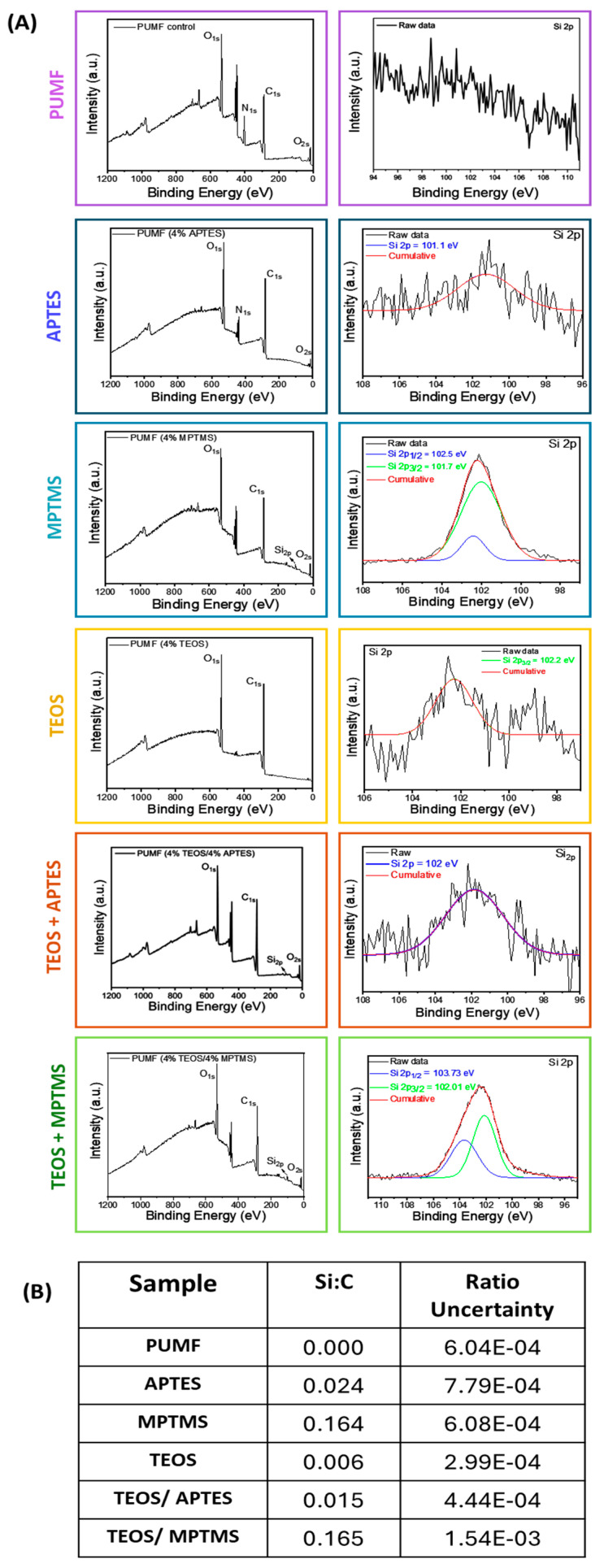
X-ray photoelectron spectroscopy (XPS) of PUMF-untreated and -functionalized microcapsules. To mitigate charge buildup during photoemission, the microcapsules were gently pressed onto indium foil. Measurements were performed using monochromated Al Kα radiation (1486.6 eV), with survey spectra acquired at pass energy of 100 eV and fine-scan spectra at 35 eV. (**A**) Survey spectra of PUMF control and functionalized microcapsules in the energy range from 0 to 1220 eV, and high-resolution spectra of the Si 2p region, from 100 to 96 eV. (**B**) Normalized silicon/carbon ratios (Si:C) derived from integrated areas under C 1s and Si 2p peaks, as well as the degree of uncertainty of the acquired results, for all tested groups.

XPS was employed to analyze the surface composition and chemical states of the samples, with the results presented in Figure 6. For all experimental groups, an initial survey spectrum was acquired spanning binding energies from 0 to 1200 eV. Signals corresponding to O 1s, N 1s, C 1s, Si 2p, and O 2s were observed, indicating the presence of these elements. The absence of peaks above 1000 eV confirms that no elements with higher atomic numbers were detected. As anticipated, a high intensity of the N 1s signal was observed across all groups, attributed to the urea and melamine content of the polymer microcapsule shell. Notably, the intensity of the N 1s signal decreased significantly in all functionalized samples. Given that the XPS detection depth is approximately 5 nm [43], any additional coating on the microcapsules would obscure the surface, leading to a decrease in N 1s intensity. This serves as a strong preliminary indication that surface modification was successful for all the groups. The reduction in N 1s intensity was relatively lower in the APTES-containing groups, which is expected due to the amino terminal group of APTES molecule. All functionalized groups exhibited an increased signal at 101.7 eV, corresponding to Si 2p [43], confirming the successful deposition of silane on the microcapsule surfaces. High-resolution spectra were acquired in the binding energy range of approximately 110 to 96 eV to further investigate silane deposition. Due to limited peak resolution, specific binding energies could not be precisely assigned, but approximations were made using peak fitting. Si:C ratios were calculated based on the areas under the C 1s and Si 2p peaks, and the results ranged from 0 to 0.165 (Figure 6B). Higher Si:C ratios indicate a greater enrichment of Si on the microcapsules surface. The results indicated that while Si 2p signals were detected for all groups, the intensity was significantly lower for APTES-containing groups. This is likely because the silanized monolayer deposited by APTES has an average thickness of 0.5 to 0.8 nm [44,45,46,47], and with an XPS scan depth of approximately 5 nm, the signals from more abundant elements such as O 1s and C 1s would overshadow the Si 2p signal. The MPTMS and TEOS/MPTMS groups showed the most intense Si 2p peaks, as evidenced by the increased Si:C ratios of 0.164 and 0.165, respectively. The observed increase in silane deposition for these groups is likely attributable to differences in the hydrolysis kinetics of MPTMS, APTES, and TEOS. During the initial hydrolysis of these silane agents, the reaction rate is influenced by the size and nature of the alkoxy-leaving groups [37]. MPTMS, which contains methoxy groups, undergoes hydrolysis more rapidly compared to TEOS and APTES, which have ethoxy groups. The ethoxy groups introduce greater steric hindrance, thereby slowing the hydrolysis process. As a result, MPTMS is more readily available for condensation onto the microcapsule surface, leading to increased silane deposition. Apart from the PUMF control, the TEOS group exhibited the lowest Si:C ratio, which is likely related to the lack of electron-donating substituents, which decrease hydrolysis rates in acidic media, as discussed previously [37]. The degree of uncertainty or error was calculated for all groups, ranging from 1.54 × 10^−3^ to 7.79 × 10^−4^ corresponding to 0.009% and 0.03%, respectively. Since these results fall below 10%, it is reasonable to conclude that they accurately reflect the surface composition of the microcapsules.

**Figure 7:**
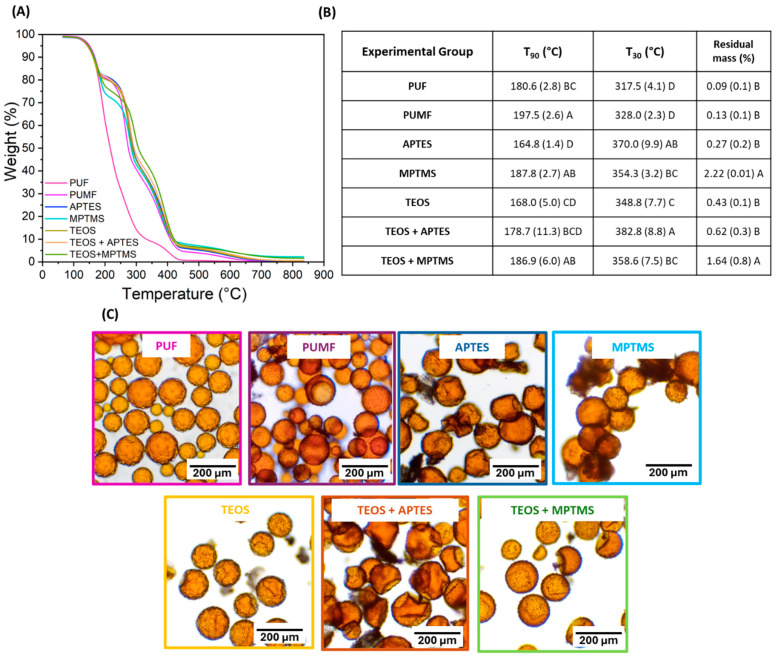
(**A**) Thermogravimetric curves of unfunctionalized and functionalized microcapsules with DHEPT incorporated into the healing agent. Analyses for each group were collected in triplicate, averaged, and the thermogram was plotted. (**B**) Table depicting T90 (temperature at which the samples lost 10 wt% of their mass), T30 (temperature at which the samples lost 70% of their mass), and residual mass after the test, which accounts for the silica formation that is not thermally decomposed at temperatures up to 850 °C. (**C**) Optical micrographs of all tested microcapsule groups, taken immediately after washing and drying, at 10× magnification.

In the final phase of this study, the aim was to evaluate the impact of the shell functionalization treatments on the mechanical performance of microcapsules incorporated into a dental resin formulation. This is a key aspect, as these microcapsules are designed to endow synthetic polymeric systems with self-healing capabilities. Consequently, all tested systems were resynthesized with the addition of DHEPT as a proton donor as part of the composition of the healing agent in the microcapsules core to enable its redox polymerization upon contact with BPO dissolved in the monomer matrix. Perhaps expectedly, the incorporation of DHEPT impacted the reaction efficiency as well as the thermal and physical properties of PUMF microcapsules. The percentage yield of PUMF microcapsules decreased from 66.4% to 43.46%. Regarding TGA, while both non-DHEPT and DHEPT-containing capsules exhibited similar mass loss events, the thermogram profiles changed significantly (Figure 5 and Figure 7), as highlighted by the T_90_ and T_30_ results. The negative impact of DHEPT incorporation on the properties of the microcapsules is likely due to the innate raise in the pH of the reaction medium resulting from the addition of an amine, consequently affecting the thermopolymerization reaction of the microcapsule shells. It has been shown that the urea–formaldehyde condensation reaction is highly dependent on pH conditions, with acidic conditions (pH < 3.5) being more favorable [48]. The first phase of the urea–formaldehyde reaction is the methylolation step, where formaldehyde reacts with urea, a process that inherently slows down at higher pH levels [48,49]. In the subsequent phase, the condensation polymerization reaction occurs, where methylolureas, additional urea molecules, and residual free formaldehyde react to form polymeric chains [48]. This reaction step is initiated by creating an acidic environment [49]. It has been demonstrated that an acidic pH catalyzes the formation of urea–formaldehyde networks, while basic conditions slow down the reaction and reduce its efficiency [48,49]. This is related to the fact that, under acidic conditions, formaldehyde is more likely to exist in a protonated form, which is more reactive towards nucleophilic attack by urea [49]. This accounts for the lower percentage reaction yield observed in this study when DHEPT was added. Furthermore, in the reaction medium, the interaction between urea and formaldehyde leads to the formation of linear and branched polymers, as well as crosslinked chains [50]. When the reaction is slower and the environment less reactive, it is reasonable to assume that branched and linear polymeric networks will predominate over crosslinked ones, impacting the physicochemical properties of the shells and, consequently, their thermal behavior. Furthermore, at pH values greater than 3.5, an increase in UF nanoparticles, which do not deposit onto the microcapsule surface, has been observed, resulting in an inefficient reaction and weaker overall capsules [51]. It is important to note that the synthetic steps for the PUF and PUMF microcapsules followed protocols widely described in the literature, where the reaction pH is adjusted to mildly acidic conditions of 3.5 after the addition of the shell starting materials [10,11,52]. Optimization of these synthetic steps may involve readjusting the pH when the healing agent containing DHEPT is added to form the microemulsion. This will be tested in a separate study. Despite the unexpectedly significant impact of DHEPT incorporation on the microcapsule synthetic reactions, it was found that functionalization of the microcapsules resulted in a right shift of the thermal degradation curves for all experimental groups, indicating that the surface modification treatments were successfully performed (Figure 7A,B). In addition, the resulting microcapsules showed structural integrity and successfully retained their cargo, as evidenced by the optical micrographs (Figure 7C). Therefore, the microcapsules were incorporated into a dental resin matrix, and the healing performance of the microcapsule-containing materials was assessed.

**Figure 8:**
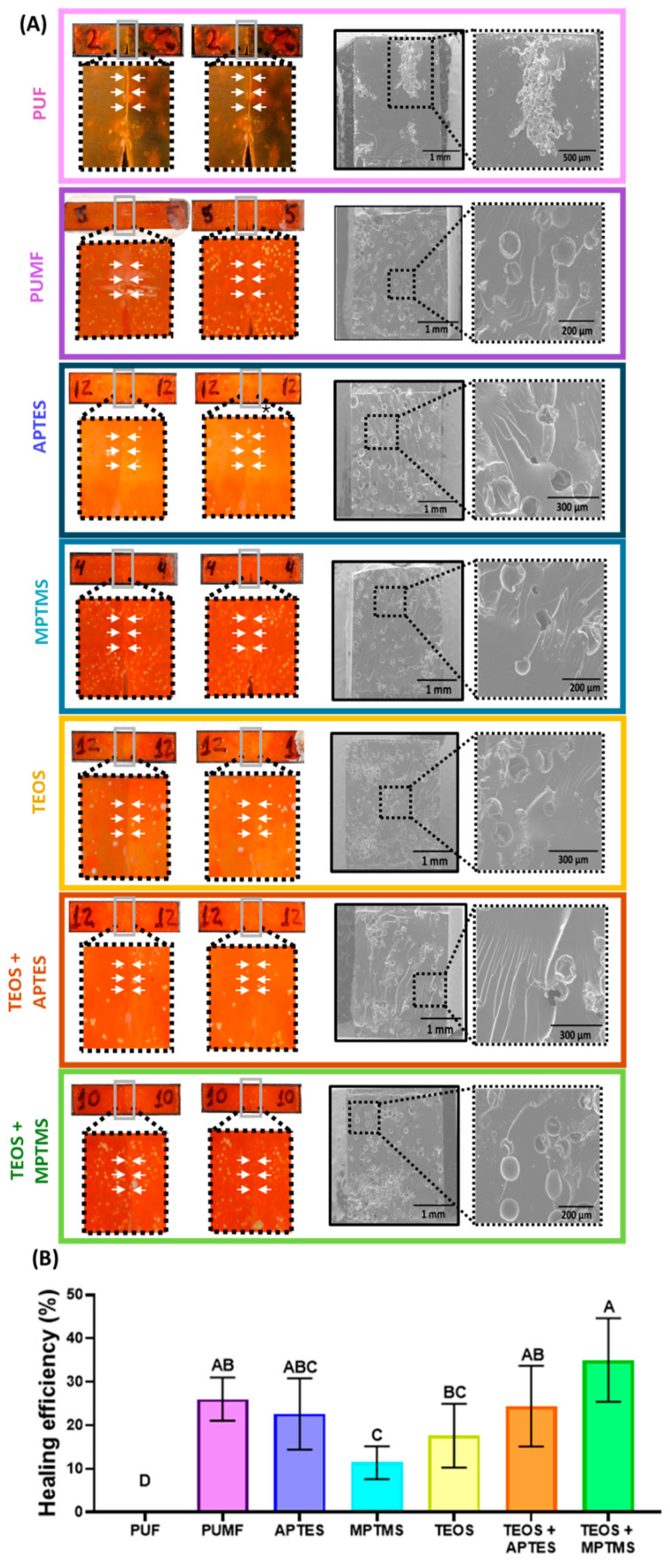
(**A**) Photographs and SEM micrographs of the fracture surface of representative fracture toughness bars composed of BisGMA, BisEMA, UDMA, and TEGDMA containing 10% PUMF-functionalized or control PUF and untreated PUMF microcapsules. The photographs show the fractured area adjacent to the notch immediately after the initial fracture (left side) and after 24 h of healing at 37 °C (right side). (**B**) Healing efficiency for all tested groups, calculated based on fracture toughness results from the initial fracture and after healing, with values ranging up to 35%. Data from all groups were analyzed by one-way ANOVA and Tukey’s test; different letters indicate statistically significant differences (*p* < 0.05).

The healing efficiency percentage, photographs of representative samples immediately post-fracture induction and after a 24 h healing process, along with SEM micrographs of the fractured surfaces for all tested groups, are presented in Figure 8. Overall, all experimental groups showed a statistically significant improvement in healing efficiency compared to the unmodified PUF control, which exhibited minimal recovery. In comparison to the other tested groups, PUF capsules are unevenly distributed and tend to form large aggregates. These aggregates remain largely undisturbed during the crack propagation, resulting in minimal–no healing agent release. The situation is exacerbated by the fact that these microcapsules are not bonded to the organic matrix. Therefore, the clusters generate a large non-bonded interface surface area, creating a pathway for cracks to deflect away from the microcapsules, consequently preventing their rupture and the initiation of the repair process, as evidenced by the SEM micrograph (Figure 8A). Fractographic analyses of resin composite fracture areas have shown that interfaces are a common propagation path for microcracks, as it is the weaker area [53,54]. In this study, inorganic filler particles were deliberately excluded from the dental resin formulations to better evaluate the interaction between the microcapsule shell and the organic matrix. The matrix composition, consisting of BISGMA, BisEMA, UDMA, and TEGDMA, was chosen for its high toughness [55,56]. The inclusion of inorganic fillers, however, would likely increase the brittleness of the system and promote crack propagation towards the microcapsules. This is likely the reason for the lower healing efficiency percentages observed in this study compared to those reported in the literature [10,11,57]. Furthermore, the formulations tested in this study possess significantly higher toughness and mechanical properties. For instance, our systems start with an elastic modulus of around 4.8 GPa, compared to 2.0 GPa in the BISGMA-TEGDMA systems [10,11,57,58], presenting a much greater challenge for the microcapsules in restoring mechanical properties after a catastrophic fracture. Another interesting finding of this study is that untreated PUMF microcapsules exhibited high healing efficiency, outperforming even some of the chemically modified surface groups. This is likely due to the increased roughness of their surface caused by the adhesion of melamine–formaldehyde nanoparticles, as discussed earlier. The increased surface roughness results in a higher surface area, which can create mechanical interlocking as the organic matrix penetrates and polymerizes in situ [15]. Given the low viscosity of the organic matrix, favored by the absence of filler particles, this penetration is further facilitated. Therefore, it is plausible to assume that mechanical interlocking was highly efficient in stabilizing the microcapsules and directing crack propagation through them, leading to their rupture and triggering the healing process. This assumption is supported by SEM micrographs of the fracture area, which show broken capsules and polymer films (Figure 8A).

**Figure 9:**
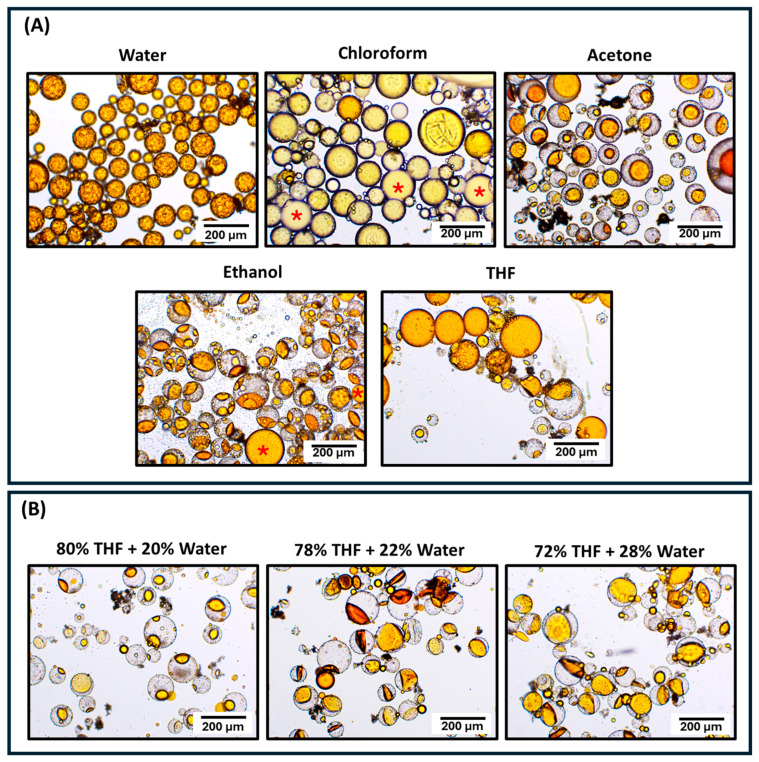
(**A**) Optical micrographs of untreated PUMF microcapsules at 10× magnification after 24 h of incubation of 200 mg of the microcapsules in 2 mL of organic solvents with varying polarities for 1 h. These solvents were evaluated for their potential to dissolve thiourethane and simulate conditions for functionalization procedures, aiming to assess the morphological integrity of the microcapsules and preservation of the healing agent volume. Water was tested as a control, as functionalizing solutions typically require some water percentage to promote hydrolysis and formation of silanol groups. Red asterisks indicate aggregation of the leaked healing agent observed in the groups treated with chloroform and ethanol. (**B**) Since tetrahydrofuran (THF) was identified as the most promising solvent, it was mixed with water in concentrations ranging from 20% to 28% by weight to simulate conditions required for functionalization procedures over 24 h. The micrographs reveal a significant loss of the healing agent, rendering this solvent system unsuitable for further functionalization.

MPTMS- and TEOS-functionalized microcapsules demonstrated significantly lower healing efficiency compared to untreated PUMF microcapsules. The suboptimal performance of MPTMS-functionalized microcapsules may be attributed to the thicker coating along with the greater flexibility introduced by thiol–carbon bonds. The increased thickness of the deposited silane layer is evidenced by the XPS results, which show the highest Si:C ratio concentration for this group. This thicker layer likely hindered the penetration of the organic matrix and reduced mechanical interlocking. In addition, while the pendant thiols in the MPTMS group are intended to chemically react with the C = C bonds in the methacrylate-based organic matrix through thiol-ene polymerization, it has been shown that the introduction of flexible thiol–carbon bonds in methacrylate-based polymeric networks can compromise mechanical properties [59,60]. Consequently, it is plausible to assume that the weakened organic matrix directed microcrack propagation through the matrix by offering low resistance and not causing the rupture of the microcapsules, thereby preventing the release of the healing agent. It is important to mention that the original strategy was to functionalize the microcapsules with thiourethanes to prevent the negative effects on the mechanical properties, but the attempts were unsuccessful due to the solvent system required to dissolve the thiourethane oligomer in the functionalizing solution. In fact, PUMF microcapsules were incubated in chloroform, acetone, ethanol, and tetrahydrofuran (THF) for 1 h, and their structural morphology and healing agent volume were analyzed via optical microscopy (Figure 9A). These solvents, with polarities ranging from 1.0 to 2.88 D, were selected for their ability to dissolve thiourethane oligomers. Among these, THF was most effective in preserving the integrity of the microcapsules. However, when THF was mixed with water at concentrations ranging from 20% to 28%, which is sufficient to promote hydrolysis and formation of silanol groups without inducing thiourethane precipitation, extensive leakage of the healing agent was observed (Figure 9B). Regarding the performance of TEOS-modified microcapsules, it was anticipated that this group might not be capable of creating efficient chemical bonding with the organic matrix. TEOS lacks additional functional groups beyond the four ethoxy groups. In fact, the functionalization with TEOS was proposed to reinforce the mechanical properties of the shell and tentatively increase the hydroxyl groups, which are prone to react with APTES and MPTMS. The single-TEOS-functionalized group was tested as a control to decouple the effects of the bilayer treatments. The lower performance of TEOS-modified microcapsules, compared to untreated PUMF microcapsules, may be related to changes in the physicochemical properties of the microcapsule shell. These changes could reduce the surface energy of the microcapsule surface, thereby preventing the organic matrix from penetrating and forming the necessary mechanical interlocking.

Interestingly, the combination of these two treatments with the lowest individual performance resulted in the surface modification that generated the highest numerical healing efficiency (TEOS/MPTMS healing recovery = 35%). This group, along with the MPTMS-only treatment, exhibited the highest Si:C ratio values. Although the TEOS coating did not significantly increase the silane deposition, as evidenced by the similar Si:C ratios between the MPTMS and TEOS/MPTMS treatments as shown by XPS analysis, the pre-treatment with TEOS appears to have mitigated the deleterious effects of MPTMS on the mechanical properties of the organic matrix. This pre-treatment likely facilitated crack propagation towards the microcapsules, resulting in their rupture and subsequent healing agent release, as demonstrated by the SEM micrographs for this group showing extensive polymer films. This phenomenon may be attributed to two main reasons: (1) hydrogen bond formation between the terminal thiols from MPTMS and silanol groups from other TEOS molecules and (2) pronounced structural deformation of the microcapsules. In the first case, hydrogen bond formation by the terminal thiol groups may have decreased the formation of thiol–carbon bonds to a level sufficient to chemically bond the microcapsule shell and the organic matrix without compromising the bulk properties of the material [61,62,63]. Additionally, these hydrogen bonds may have reinforced the coating stability and increased the surface energy of the microcapsule surface, improving wettability and thus enhancing the penetration of the organic matrix through the undercuts and creating an efficient mechanical interlocking [61,62,63]. Furthermore, the capsules subjected to the bilayer treatment exhibited greater morphological deformation than those subjected to a single treatment (Figure 7B). This is mainly due to extended exposure to solvents and their subsequent evaporation [13]. This change in conformation may also lead to better interlocking between the organic matrix and the microcapsules, helping to stabilize them and preventing microcracks from deflecting away.

**Figure 10:**
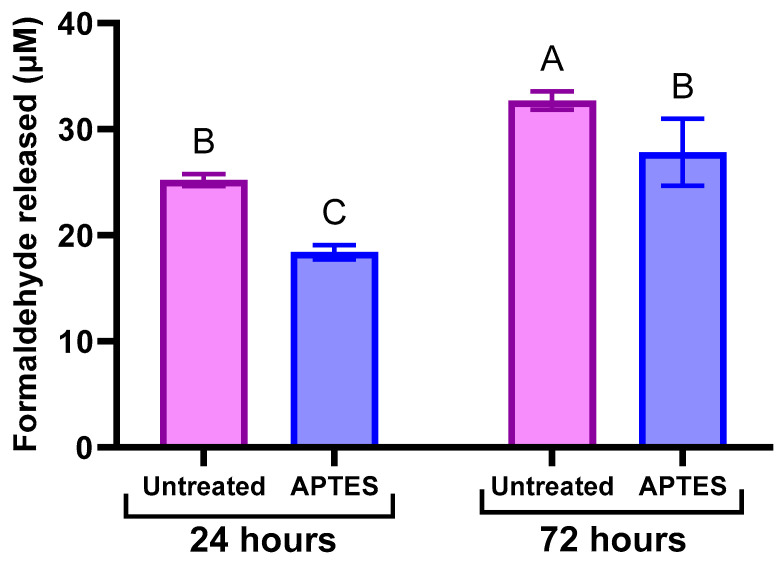
Average formaldehyde release (in µM) quantified by Purpald colorimetric assay for both untreated PUMF and APTES-functionalized microcapsules incubated in distilled water for 24 h and 72 h. Briefly, 100 mg of microcapsules were suspended in 5 mL of 1X PBS and then placed in 12–16 kDa molecular weight cut-off (MWCO) dialysis tubing, which was sealed. The dialysis tubing was immersed in 100 mL of 1X PBS and placed on a shaker operating at 60 RPM [29]. The samples were incubated at 37 °C, and aliquots of the outer PBS solution were collected at 24 and 72 h. Separately, a 34 mM Purpald solution in 2 M NaOH and a 2 mM sodium periodate solution in 0.2 M NaOH were prepared. For the assay, 0.5 mL of each PBS aliquot was mixed with 0.5 mL of Purpald solution and incubated at room temperature for 20 min. After incubation, 0.5 mL of sodium periodate solution was added to develop and stabilize the color [29]. Measures of 200 µL of each developed solution was transferred to a 96-well plate, and absorbance at 560 nm was measured using a plate reader. Formaldehyde concentrations were determined using linear regression from a calibration curve with formaldehyde concentrations of 50, 5, 0.5, and 0.05 µM. Data were analyzed using two-way ANOVA followed by Tukey’s test, with different letters indicating statistically significant differences (*p* < 0.05). The results demonstrated that surface modification with APTES significantly reduced overall formaldehyde emission, confirming its effectiveness as a formaldehyde scavenger.

Regarding APTES performance, its association with TEOS coating did not result in any significant change in terms of healing efficiency, and both APTES and TEOS/APTES groups showed results similar to the PUMF control. APTES is a common functionalizing agent with amino terminal groups and has been used for various purposes, such as imparting corrosion resistance on metal surfaces [21]. This silane was selected for this study due to the possibility of amino groups reacting with methacrylate groups to create β-amino ester-based networks [17], and because the terminal amino groups can act as formaldehyde scavengers, significantly limiting its emission [16]. The presence of formaldehyde in microcapsules designed for self-healing dental polymers has raised concerns about potential toxic effects. Although comprehensive studies exploring this issue are not yet available in the literature, it is reasonable to assume that some release of formaldehyde may occur. To confirm the formaldehyde scavenging capability of APTES, untreated and APTES-functionalized microcapsules had their formaldehyde emission assessed using Purpald colorimetric assay [29] (Figure 10). The results demonstrated a significant reduction in formaldehyde emission from APTES-functionalized microcapsules at both the 24 h and 72 h time points compared to untreated PUMF microcapsules (Figure 10). This underscores the effectiveness of APTES functionalization as a viable strategy for mitigating the intrinsic formaldehyde content in PUF-based microcapsules by acting as a scavenger.

## 4. Conclusions

In this study, innovative surface modifications for microcapsules intended for self-healing resin-based dental materials were developed and evaluated. A novel approach to functionalization for PUMF microcapsules was introduced, utilizing advanced silanizing agents such as APTES and MPTMS, and pioneering bilayer functionalization protocols through their combination with TEOS were presented. Reinforcing PUF microcapsules with melamine significantly enhanced their durability during the functionalization procedures, which involved additional mechanical and chemical stresses. Various chemical modifications of the microcapsule surfaces led to differing performance outcomes. Overall, all surface-modified microcapsules demonstrated improved healing efficiency compared to the control PUF capsules, with an average increase ranging from 15 to 35 wt%. This enhancement is attributed to their more uniform integration into the resin and better interfacial bonding with the organic matrix. Notably, the combination of TEOS and MPTMS resulted in superior healing efficiency, achieving approximately 35% of the original toughness. This improvement is likely due to TEOS incorporation, which mitigated the adverse effects of thiol–carbon bond formation within the organic matrix. APTES-modified capsules exhibited 28% lower formaldehyde emissions compared to untreated capsules, potentially enhancing the biocompatibility of these systems. In summary, surface functionalization emerges as a promising approach for improving interfacial adhesion between microcapsules and dental resin matrices. Selective modification of microcapsules for self-healing applications can further enhance healing recovery in fractures and offers a valuable strategy for tailoring microcapsules to meet specific application needs. Future studies will focus on incorporating the newly synthesized microcapsules into dental resin composite formulations, tracking microcrack formation and healing using dyes, and conducting an in-depth analysis of the biological impact of the chemical modification treatments.

## Data Availability

The data presented in this study are available on request from the corresponding author.
